# Bridge-Work

**Published:** 1894-06

**Authors:** C. M. Richmond

**Affiliations:** New York.


ARTICLE VII.

### BRIDGE-WORK.

DR. C. M. RICHMOND, NEW YORK.

In making bridge-work, I may say that I have in my practice abandoned complications which appear to me to be fatal to this operation as a practical operation. I have reduced the practice to the simplest possible method, demonstrating to men who have visited my office ideas of bridge-work which to them have been a revelation. I discovered accidentally that I could make a band and drive it to what we term the largest or bulging portion of the tooth, and not beyond it, where it would make an imperfect condition of things by driving the band over a surface where it could not fit, with the margin of the band under the gum, where we could not get at it. Those bands have been cemented on for eight years, over two teeth supporting an upper portable bridge. That case convinced me that it was unnecessary to carry the band to the neck of the tooth. That relieved me of the necessity of cutting down the tooth. A crown can be fitted over a tooth, leaving the gum-line entirely free, so that the brush will keep it clean without the slightest difficulty. In regard to fitting the abutments, this piece of mechanism is very commendable. In fastening portable bridges, I make gold crowns which are cemented on as abutments, and then I make bands which are made of clasp metal, always using whatever methods are best for the case, for keeping the band from slipping farther than the required point. I find by making those bands of clasp metal that they never bend or stretch, because they fit tightly.

I have worn in my own mouth, for eight years, bridge-work with three attachment points only. That case has been removed every day, cleansed, and replaced, and it goes on exactly as tight to-day as it did when I commenced to wear it. Those band-attachments are made of clasp metal, and the only point of attachment, the end of the root, is covered with a gold crown, and in that gold crown is a tapering, square tube, which fits accurately when it is home. It is quite loose till it arrives at the point where it was made to fit; the moment it reaches that point it is firm. It starts very hard, but when it is started from its position it very readily comes off. Two roots and two bands of that description will hold an entire upper set rigid. The patient can take it off every day and cleanse it without the danger of its getting loose. The idea of having these fastened to the teeth, one at a time, seems to me practically perfect The dentist can fasten one tooth with cement, and get it secure every time; but when he has to fasten on five or six crowns, that is a different thing. I have never seen an entire case where every one of the bands was perfectly cemented. I have found one, two, three, or four perfect ones, but one or two crowns were imperfectly cemented. Therefore, portable bridge-work can be fastened to one tooth and perfectly cemented, but when attached to four or five teeth it cannot be done.—*Cosmos.*

